# Author Correction: Fast and multiplexed superresolution imaging with DNA-PAINT-ERS

**DOI:** 10.1038/s41467-020-18724-x

**Published:** 2020-09-21

**Authors:** Fehmi Civitci, Julia Shangguan, Ting Zheng, Kai Tao, Matthew Rames, John Kenison, Ying Zhang, Lei Wu, Carey Phelps, Sadik Esener, Xiaolin Nan

**Affiliations:** 1 Knight Cancer Early Detection Advanced Research Center, Oregon Health and Science University, 2720 S. Moody Ave., Portland, OR 97201 USA; 2 Center for Spatial Systems Biomedicine, Oregon Health and Science University, 2730 S. Moody Ave., Portland, OR 97201 USA; 3 Department of Biomedical Engineering, Oregon Health and Science University, 3303 S. Bond Ave., Portland, OR 97239 USA; 4 Department of Oral Maxillofacial-Head Neck Oncology, School and Hospital of Stomatology, Wuhan University, 237 Luoyu Rd., Wuhan, 430079 Hubei China

**Keywords:** Super-resolution microscopy, Single-molecule biophysics

Correction to: *Nature Communications* 10.1038/s41467-020-18181-6, published online 28 August 2020.

In the original version of this Article, the DOI for Reference 21, describing protocols for sample preparation and imaging procedures used in this Article, is incorrectly cited as 10.1038/protex.2011.204. The DOI link should be 10.21203/rs.3.pex-1069/v1. This has been corrected in both the PDF and HTML versions of the Article.

