# Supplementary material for: Fast and multiplexed superresolution imaging with DNA-PAINT-ERS
Source: Nat Commun. 2020 Aug 28;11:4339. doi: 10.1038/s41467-020-18181-6 (PMC7455722; doi:10.1038/s41467-020-18181-6)
Supplement: Supplementary file 1 — Supplementary Information [file 41467_2020_18181_MOESM1_ESM.pdf]

Fast and multiplexed superresolution imaging with DNA-PAINT-ERS

Civitci *et al.*

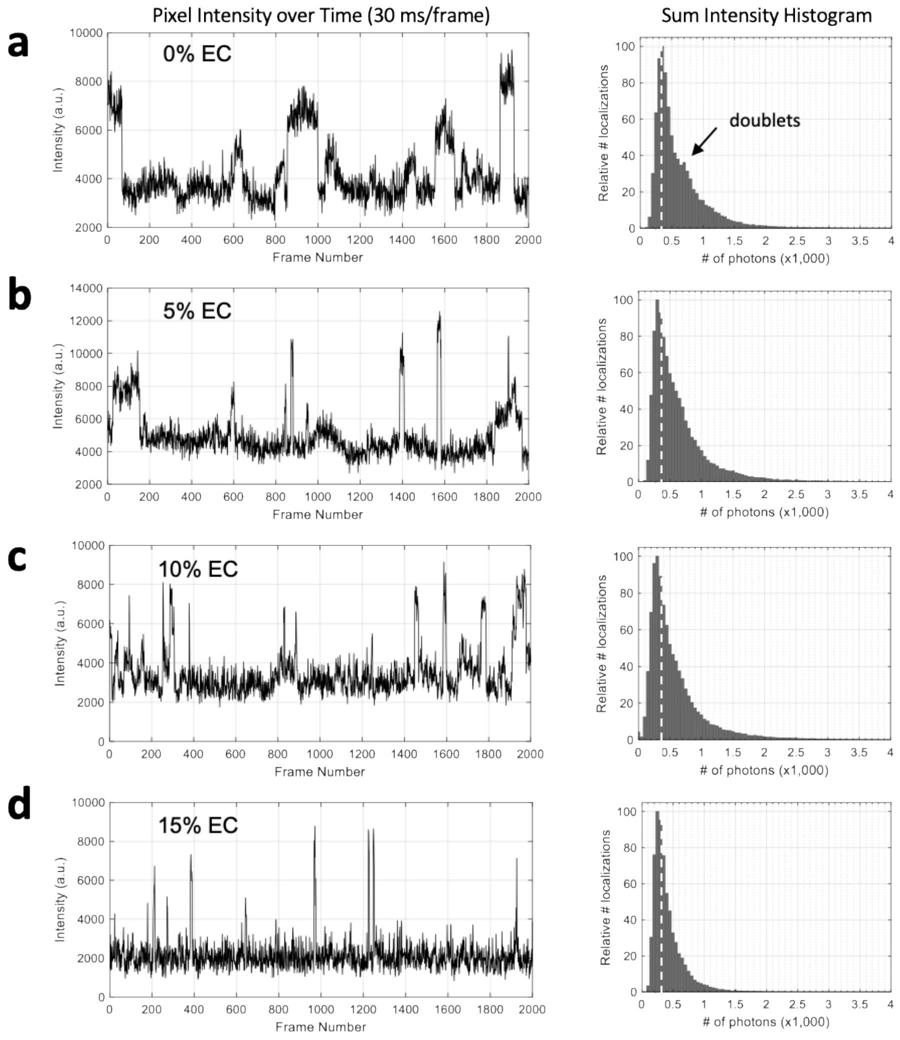

Supplementary Figure 1. Effect of EC on the localization kinetics of the DS1-IS1 pair. The plots on the left represent the intensity fluctuations at a single pixel in the raw image stacks acquired at 30 ms per frame at 0% (a), 5% (b), 10% (c), and 15% (d) EC. Shown on the right are histograms of sum intensities for 0.5 – 1 million raw localizations in each case. or a localization event, the sum intensity was computed as $2\pi\sigma_x\sigma_y a$ based on Gaussian fitting results, where σ_x and σ_y are the half-widths of the point spread function in the x and y dimensions, respectively, and a is the fitting amplitude. The dotted line in the histograms mark the peak position in the 1st histogram (at 0% EC). Note the shoulder peak in the first histogram likely due to doublets (two localizations appearing at overlapping pixels), which became much less prominent at higher EC%. Images were taken with a laser power density at ~100 W cm^{-2} to reduce photobleaching and reveal true localization kinetics.

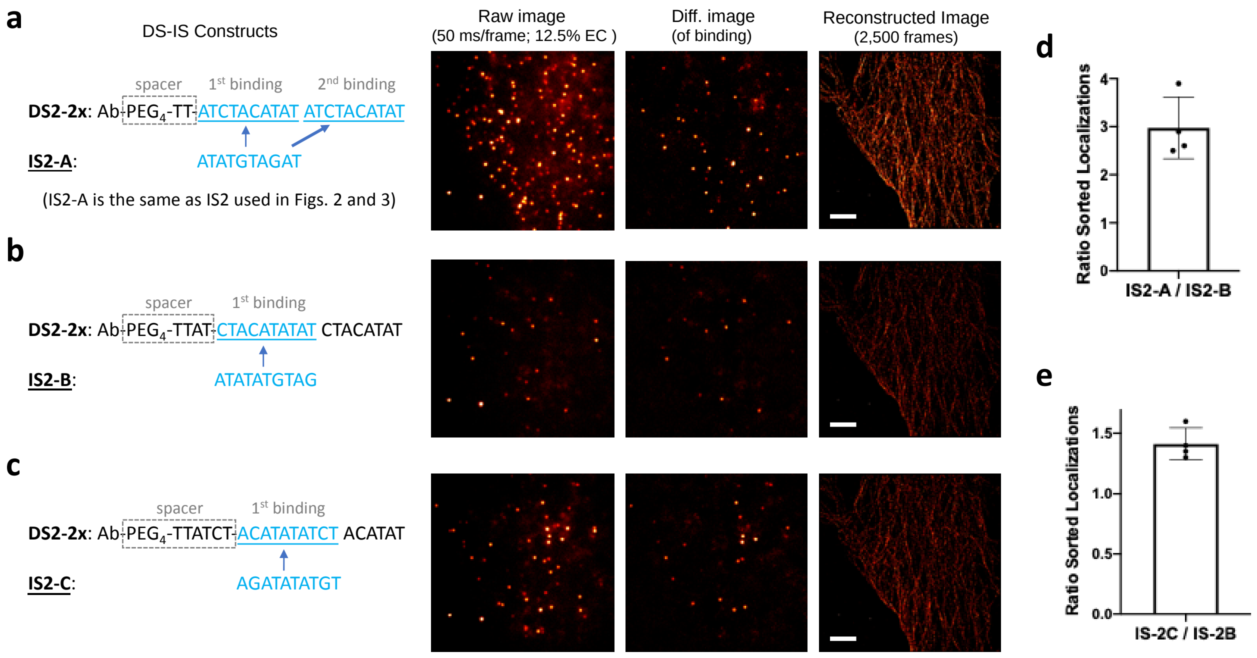

Supplementary Figure 2. Effect of repeating sequence and spacer length on the localization kinetics using DS2-2x-PEG4. Three distinct IS oligos, namely IS2-A, IS2-B, and IS2-C, bind to 2 sites (a, left), 1 site (b, left), and 1 site (c, left), respectively, on DS2-2x-PEG4. In the DS2-2x-PEG4 construct, PEG4 and the non-complementary bases between the antibody (Ab) and the 1st IS-binding sites could be viewed as spacers. U2OS cells were fixed, permeabilized, and immuno-labeled for microtubules using an anti-Rabbit primary and a rabbit secondary antibody conjugated to DS2-2x-PEG4; the sample was subsequently imaged with DNA-PAINT in buffer C containing 12.5% EC using 2 nM CF660R conjugated IS2 (-A, -B, or -C) and a frame acquisition rate of 50 ms per frame. Each field of view was imaged using the three IS oligos in three sequential cycles, and the sample was carefully and thoroughly washed in between the cycles. The resulting raw images (a-c, 2nd column), differential images featuring binding events (a-c, 3rd column), and resulting DNA-PAINT reconstructions (from 2,500 raw frames; a-c, 4th column) show clear distinctions in the imaging kinetics depending on the IS used. The ratios of sorted localizations between IS-2A and IS-2B (Mean±SD: 3.0 ± 0.6) or between IS-2C and IS-2B (Mean ± SD: 1.4 ± 0.1) are shown in (d) and (e), respectively, with IS-2B used as a normalization standard. Results from 4 field of views from two independent experiments were recorded and analyzed. Scale bars, 5 µm. Error bars in (d) and (e) standard deviations. Source data underlying Supplementary Figure 2d and 2e are provided as a Source Data file.

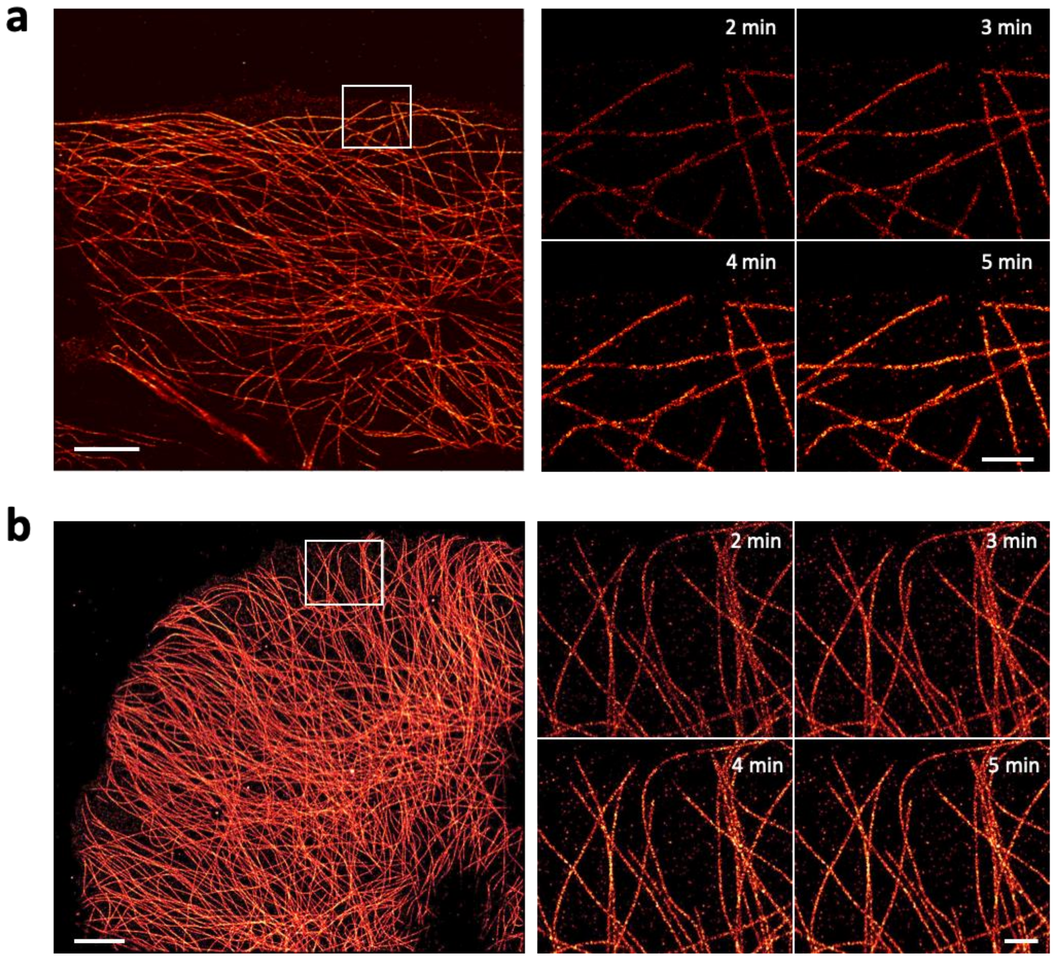

Supplementary Figure 3. DNA-PAINT-ERS imaging of microtubules in non-extracted U2OS cells. (a) and (b) are examples from two independent experiments. Cells were immunostained using a mouse anti-tubulin primary and then a goat anti-mouse secondary antibody conjugated with DS1-2x-PEG16. The cells were then imaged using 2.5 nM IS1-CF660R at 15 ms/frame. See also the raw single-molecule video for the localization dynamics (Supplementary video 4). These imaging conditions were the same as described in Fig. 3a. In both (a) and (b), the left panel shows the overall structure of a cell; middle panel shows zoom-in views of the reconstructed structures in the boxed area. Similar results were obtained from six cells in two independent experiments. Scale bars, 5 μm (left, a & b), 1 μm (right, a & b).

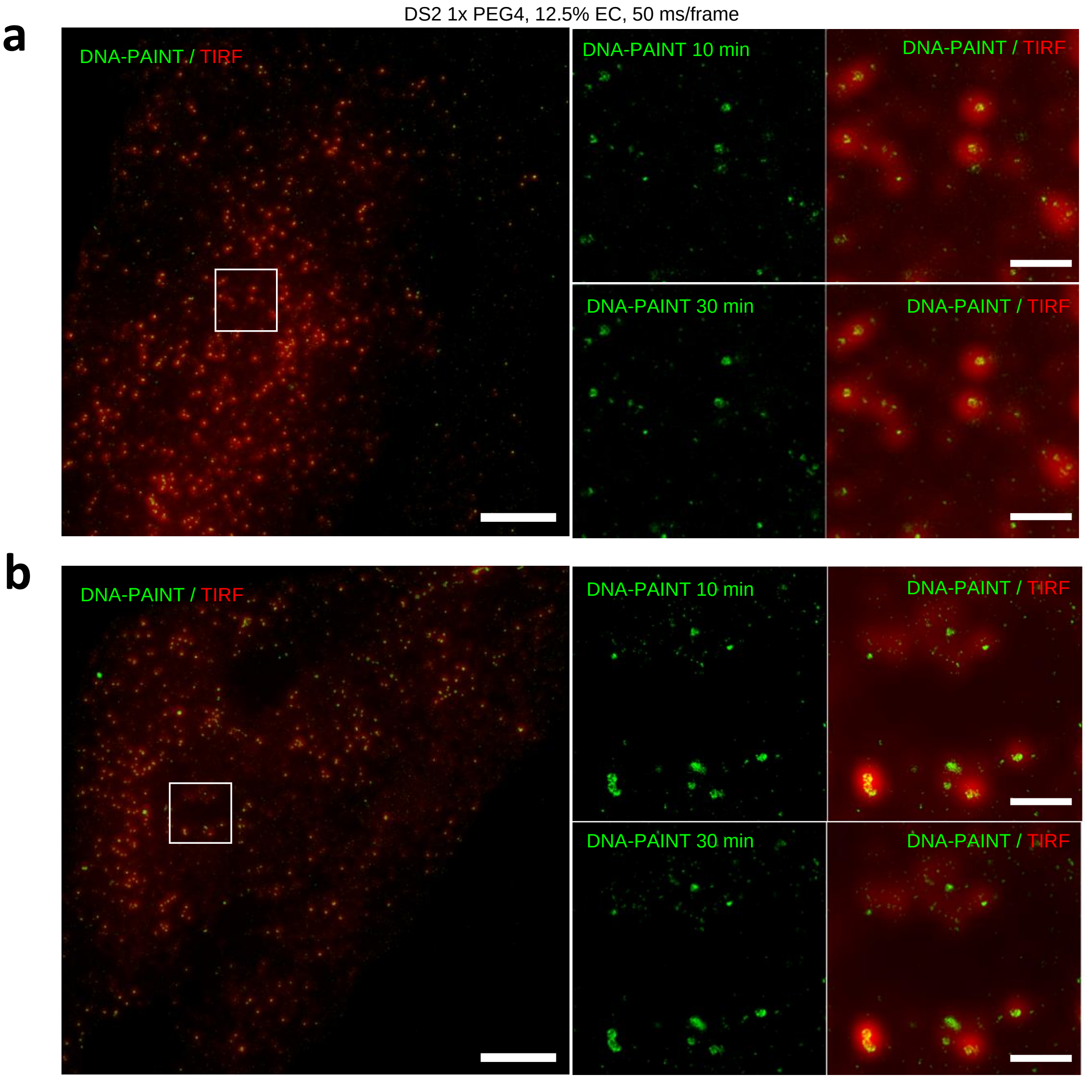

Supplementary Figure 4. DNA-PAINT imaging of caveolae using DS2 (1x) with a PEG4 spacer. Images shown in (a) and (b) are from two independent experiments, and the example in (a) is the same as shown in Fig. 4c (left column) with the full FOV. Left panel shows an overlaid TIRF (red) and DNA-PAINT (green) image of caveolae in the same U2OS cell, and right panel shows zoom-in views of the boxed region on the left with DNA-PAINT data taken within 10 min (12k frames, top row) and 30 min (36k frames, bottom row). Raw images were acquired at 50 ms per frame in buffer C containing 12.5% EC. Similar results were obtained from four cells in two independent experiments. Scale bars, 5 µm (left) and 1 µm (right) in both (a) and (b).

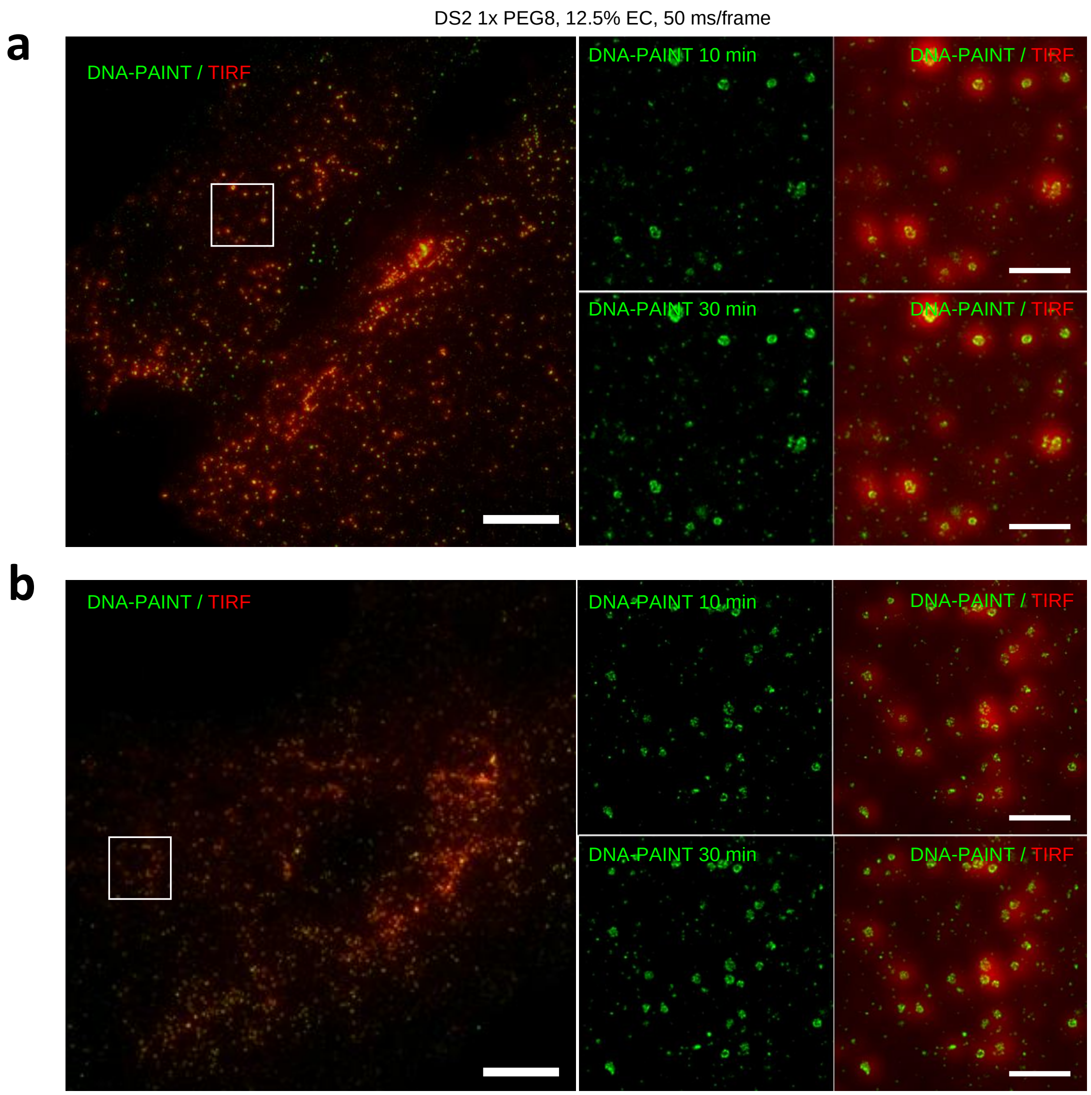

Supplementary Figure 5. DNA-PAINT imaging of caveolae using DS2 (1x) with a PEG8 spacer. Images shown in (a) and (b) are from two independent experiments, and the example in (a) is the same as shown in Fig. 4c (middle column) with the full FOV. Left panels show an overlaid TIRF (red) and DNA-PAINT (green) image of caveolae in the same U2OS cell, and right panels show zoom-in views of the boxed region on the left with DNA-PAINT data taken within 10 min (12k frames, top row) and 30 min (36k frames, bottom row). Raw images were acquired at 50 ms per frame in buffer C containing 12.5% EC. Similar results were obtained from four cells in two independent experiments. Scale bars, 5 µm (left) and 1 µm (right) in both (a) and (b).

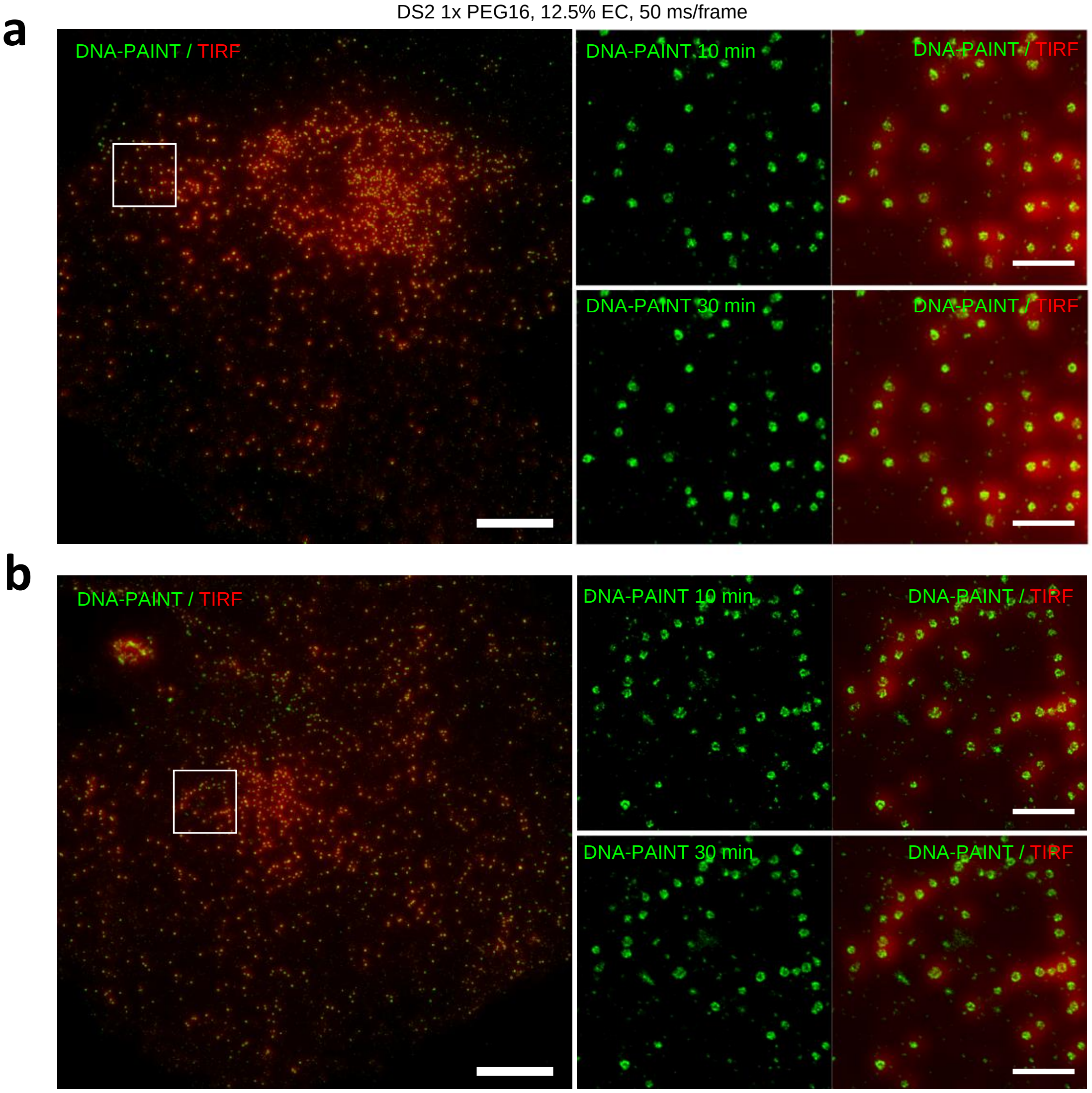

Supplementary Figure 6. DNA-PAINT imaging of caveolae using DS2 (1x) with a PEG16 spacer. Images shown in (a) and (b) are from two independent experiments, and the example in (a) is the same as shown in Fig. 4c (middle column) with the full FOV. In both (a) and (b), the left panel shows an overlaid TIRF (red) and DNA-PAINT (green) image of caveolae in the same U2OS cell, and the right panel shows zoom-in views of the boxed region on the left with DNA-PAINT data taken within 10 min (12k frames, top row) and 30 min (36k frames, bottom row). Raw images were acquired at 50 ms per frame in buffer C with 12.5% EC. Similar results were obtained from four cells in two independent experiments. Scale bars, 5 μm (left) and 1 μm (right) in both (a) and (b).

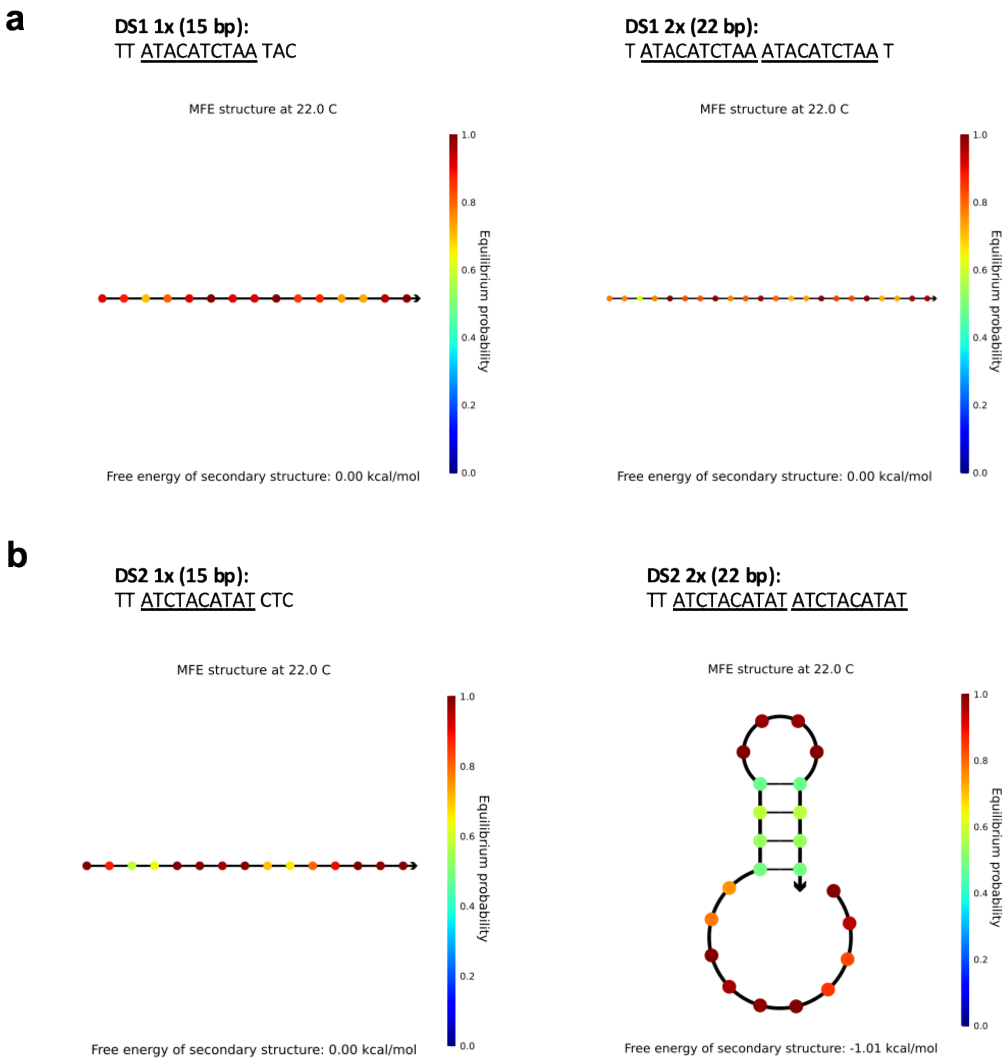

Supplementary Figure 7. Analysis of DS1 and DS2 constructs for potential secondary structures. We used NUPACK [http://www.nupack.org/partition/new] to analyze DS1-1x (a, left) and -2x (a, right) as well as DS2-1x (b, left) and -2x (b, right) constructs. All analyses were run with a temperature setting of 22 ºC.

Supplementary Table 1. Sequences of the DS-IS constructs used in the present work.*

DS	DS Sequence**	IS	IS Sequence	Tm (ºC)***	Note
**DS1**-1x	tt ATACATCTAA tac	IS1	TTAGATGTAT	12.3	Derived from P1
**DS1**-2x	t ATACATCTAA ATACATCTAA t	IS1	TTAGATGTAT	12.3	
**DS1**-3x	t ATACATCTAA ATACATCTAA ATACATCTAA t	IS1	TTAGATGTAT	12.3	
**DS2**-1x	tt ATCTACATAT ctc	IS2	ATATGTAGAT	11.4	Derived from P2
**DS2**-2x	tt ATCTACATAT ATCTACATAT	IS2	ATATGTAGAT	11.4	IS2 is the same as IS2-A
	ttat CTACATATAT ctacatat	IS2-B	ATATATGTAG	9.2	1 binding site for IS2-B on DS2-2x
	ttatct ACATATATCT acatat	IS2-C	AGATATATGT	11.4	1 binding site for IS2-C on DS2-2x

* Bases recognized by the IS in each DS construct are marked as underscored capital letters, with the rest as lower-case letters;

** Sequences complementary to that of the corresponding IS are shown as capitalized, and each unit of complementary (docking sequence) is underlined separately, and the bolded sequences in DS1-1x and DS2-1x constructs correspond to the complementary sequences in P1 and P2, respectively;

*** Tm was calculated using Genescript's online analysis tool [https://www.genscript.com/tools/oligo-primer-calculation] assuming standard buffer conditions and neglecting 5'- or 3'-modifications.
