# Supplementary material for: Fast and multiplexed superresolution imaging with DNA-PAINT-ERS
Source: Nat Commun. 2020 Aug 28;11:4339. doi: 10.1038/s41467-020-18181-6 (PMC7455722; doi:10.1038/s41467-020-18181-6)
Supplement: Supplementary file 3 — Description of Additional Supplementary Files [file 41467_2020_18181_MOESM3_ESM.pdf]

Description of Additional Supplementary Files

File Name: Supplementary Movie 1
Description: **Effect of EC on the localization kinetics of DNA-PAINT.** Video demonstrating the concentration-dependent effect of EC on the localization kinetics during DNA-PAINT imaging of microtubules in U2OS cells using the DS1 (1x) – IS1 pair. The image acquisition was at 100 ms per frame, and the laser power was ~100 W cm^{-2} to minimize photobleaching such that the true kinetics of DS-IS binding and unbinding could be probed. All images were also shown at the same contrast and played at the actual speed (10 frames $s\text{-}^{1}$)

File Name: Supplementary Movie 2
Description: L**ocalization kinetics of three different imager strands binding to the same docking strand.** Video showing the single-molecule localization kinetics during DNA-PAINT imaging of microtubules in extracted U2OS cells. A secondary antibody conjugated to DS2-2x-PEG4 was used for staining and DNAPAINT imaging was performed using 2 nM IS2-A-CF660R (left), IS2-B-CF660R (middle), and IS2-CCF660R in buffer C supplemented with 12.5% EC. Images were acquired at 50 ms per frame and played at the actual speed (20 frames s^{-1}). See Supplementary figure 2 for more details.

File Name: Supplementary Movie 3
Description: **Raw video for DNA-PAINT-ERS imaging of microtubules in extracted U2OS cells.** Video showing the single-molecule localization kinetics during DNA-PAINT-ERS imaging of microtubules in extracted U2OS cells. Imaging was carried out using the DS1-2x-PEG16 construct conjugated to a donkey anti-mouse secondary antibody and 3 nM IS1-CF660R with 15% EC in buffer C. Images were acquired at 15 ms per frame and played at the actual speed (67 frames s^{-1}).

File Name: Supplementary Movie 4
Description: **Raw video for DNA-PAINT-ERS imaging of microtubules in non-extracted U2OS cells.** Video showing the single-molecule localization kinetics during DNA-PAINT-ERS imaging of microtubules in nonextracted U2OS cells. Imaging was carried out using the DS1-2x-PEG16 construct conjugated to a donkey anti-mouse secondary antibody and 2.5 nM IS1-CF660R with 15% EC in buffer C. Images were acquired at 15 ms per frame and played at the actual speed (67 frames s^{-1}).

File Name: Supplementary Movie 5
Description: **Raw video for DNA-PAINT-ERS imaging of clathrin in extracted U2OS cells.** Video showing the single-molecule localization kinetics during DNA-PAINT-ERS imaging of clathrin in extracted U2OS cells. Imaging was carried out using the DS2-2x-PEG16 construct conjugated to a donkey anti-rabbit secondary antibody and 2.5 nM IS2-CF660R with 12.5% EC in buffer C. Images were acquired at 20 ms per frame and played at real speed (50 frames s^{-1}).
