# Supplementary material for: Fast and multiplexed superresolution imaging with DNA-PAINT-ERS
Source: Nat Commun. 2020 Aug 28;11:4339. doi: 10.1038/s41467-020-18181-6 (PMC7455722; doi:10.1038/s41467-020-18181-6)
Supplement: Supplementary file 9 — Reporting Summary [file 41467_2020_18181_MOESM9_ESM.pdf]

nature research

Corresponding author(s): Xiaolin Nan

Last updated by author(s): Aug 3, 2020

Reporting Summary

Nature Research wishes to improve the reproducibility of the work that we publish. This form provides structure for consistency and transparency in reporting. For further information on Nature Research policies, see Authors & Referees and the Editorial Policy Checklist.

Statistics

For all statistical analyses, confirm that the following items are present in the figure legend, table legend, main text, or Methods section.

n/a	Confirmed	
☐	☒	The exact sample size (*n*) for each experimental group/condition, given as a discrete number and unit of measurement
☐	☒	A statement on whether measurements were taken from distinct samples or whether the same sample was measured repeatedly
☒	☐	The statistical test(s) used AND whether they are one- or two-sided<br>*Only common tests should be described solely by name; describe more complex techniques in the Methods section.*
☒	☐	A description of all covariates tested
☐	☒	A description of any assumptions or corrections, such as tests of normality and adjustment for multiple comparisons
☐	☒	A full description of the statistical parameters including central tendency (e.g. means) or other basic estimates (e.g. regression coefficient) AND variation (e.g. standard deviation) or associated estimates of uncertainty (e.g. confidence intervals)
☒	☐	For null hypothesis testing, the test statistic (e.g. *F*, *t*, *r*) with confidence intervals, effect sizes, degrees of freedom and *P* value noted<br>*Give P values as exact values whenever suitable.*
☒	☐	For Bayesian analysis, information on the choice of priors and Markov chain Monte Carlo settings
☒	☐	For hierarchical and complex designs, identification of the appropriate level for tests and full reporting of outcomes
☒	☐	Estimates of effect sizes (e.g. Cohen's *d*, Pearson's *r*), indicating how they were calculated

Our web collection on statistics for biologists contains articles on many of the points above.

Software and code

Policy information about availability of computer code

Data collection	Superresolution images were acquired using the open source Micro-Manager software suite (version 1.4, from https://micro-manager.org/) and saved as OMERO TIF files.
Data analysis	Image analyses for extracting, filtering, sorting and rendering single-molecule localizations were performed using in-house Matlab scripts. The Matlab scripts have been available through our lab webpage (http://www.ohsu.edu/nan); we are in the process of depositing the code to GitHub for broader sharing and better version tracking. The rendered images were saved as TIF files for further analysis and annotations in Fiji (ImageJ 2.0.0, Java 1.8).

For manuscripts utilizing custom algorithms or software that are central to the research but not yet described in published literature, software must be made available to editors/reviewers. We strongly encourage code deposition in a community repository (e.g. GitHub). See the Nature Research guidelines for submitting code & software for further information.

Data

Policy information about availability of data

All manuscripts must include a data availability statement. This statement should provide the following information, where applicable:

- Accession codes, unique identifiers, or web links for publicly available datasets
- A list of figures that have associated raw data
- A description of any restrictions on data availability

All the data reported here will be available upon request. The raw data underlying Figs. 1B-D and supplementary Fig. 2D-E are provided as a Source Data file.

Field-specific reporting

Please select the one below that is the best fit for your research. If you are not sure, read the appropriate sections before making your selection.

☒ Life sciences ☐ Behavioural & social sciences ☐ Ecological, evolutionary & environmental sciences

For a reference copy of the document with all sections, see nature.com/documents/nr-reporting-summary-flat.pdf

Life sciences study design

All studies must disclose on these points even when the disclosure is negative.

Sample size	Sample size was chose to be >=3 for all cases with considerations on the total time needed for taking the full dataset. Larger datasets were acquired where practical. Please see individual figures for the exact sample size in each case.
Data exclusions	No data were excluded from analyses.
Replication	The experiments were replicated multiple (2+) times over a course of 3 months or longer, and the results were found to be consistent.
Randomization	The cells and field of views were chosen randomly for each group of experiments.
Blinding	Blinding was not applicable to this study because samples were not allocated into groups.

Reporting for specific materials, systems and methods

We require information from authors about some types of materials, experimental systems and methods used in many studies. Here, indicate whether each material, system or method listed is relevant to your study. If you are not sure if a list item applies to your research, read the appropriate section before selecting a response.

Materials & experimental systems

n/a	Involved in the study	
☐	☒	Antibodies
☐	☒	Eukaryotic cell lines
☒	☐	Palaeontology
☒	☐	Animals and other organisms
☒	☐	Human research participants
☒	☐	Clinical data

Methods

n/a	Involved in the study	
☒	☐	ChIP-seq
☒	☐	Flow cytometry
☒	☐	MRI-based neuroimaging

Antibodies

Antibodies used	1) Anti-beta Tubulin Mouse Monoclonal (ThermoFisher Scientific, cat: 32-2600, clone: 2 83 33, lot: UE283816, dilution 1:200)<br>2) Anti-Clathrin heavy chain Rabbit Polyclonal (abcam, cat: ab21679, lot: GR3201023-1, dilution 1:200)<br>3) Anti-Caveolin-1 Rabbit Polyclonal (abcam, ab2910, lot: GR3286682-1, dilution 1:200)<br>4) AffiniPure Donkey anti-Mouse IgG (H+L) (Jackson Immuno Research, 715-005-150)<br>5) AffiniPure Donkey anti-Rabbit IgG (H+L) (Jackson Immuno Research, 711-005-152)<br>6) DS1-conjugated Donkey anti-Mouse IgG, made in house using antibody 4) and DS1<br>7) DS2-conjugated Donkey anti-Rabbit IgG, made in house using antibody 5) and DS2
Validation	1) Tested for ICC, IF, WB. Reactivity confirmed in mouse NIH3T3 fibroblast cells, mouse testis and rat brain. 53 references. Cited in "Automatic Bayesian single molecule identification for localization microscopy" (Scientific Reports, 2016)<br>2) Tested for ICC, IF, WB, IHC-P, Flow Cyt, IHC-FoFr, IP. Reactivity confirmed in human kidney tissue and HeLa, A431, Jurkat and NIH3T3 cells. 74 references. Cited in "Automating multimodal microscopy with NanoJ-Fluidics" (Nature Communications, 2019)<br>3) Tested for ICC, IF, WB, IHC-Fr, IP, IHC-P, Dot blot. Reactivity confirmed in human lung, heart and spleen lysate, rat heart protein extract, NIH/3T3 and HeLa cells, and human cardiac tissue. 97 references. Cited in "An endocytosis pathway initiated through neuropilin-1 and regulated by nutrient availability" (Nature Communications, 2014)<br>4-7) Validations of secondary antibodies were done using samples prepared without the primary antibody, where lack of signals demonstrate the specificity of these secondary antibodies against antibodies raised in the corresponding species.

Eukaryotic cell lines

Policy information about cell lines

Cell line source(s)	U2OS (ATCC, HTB-96)

Authentication	Cell line was not authenticated.
Mycoplasma contamination	Cell line was not tested for mycoplasma contamination.
Commonly misidentified lines<br>(See ICLAC register)	No commonly misidentified cell lines were used.
